# The Anterior Inferior Cerebral Artery Variability in the Context of Neurovascular Compression Syndromes: A Narrative Review

**DOI:** 10.3390/biomedicines12020452

**Published:** 2024-02-17

**Authors:** Dawid Kościołek, Mateusz Kobierecki, Mikołaj Tokarski, Konrad Szalbot, Aleksandra Kościołek, Mikołaj Malicki, Sora Wanibuchi, Karol Wiśniewski, Michał Piotrowski, Ernest J. Bobeff, Bartosz M. Szmyd, Dariusz J. Jaskólski

**Affiliations:** 1 Medical Faculty, Medical University of Lodz, Kosciuszki St., 90-419 Lodz, Poland; dawid.kosciolek@stud.umed.lodz.pl (D.K.); mateusz.kobierecki@stud.umed.lodz.pl (M.K.); mikolaj.tokarski@stud.umed.lodz.pl (M.T.); konrad.szalbot@stud.umed.lodz.pl (K.S.); aleksandra.kryszewska@stud.umed.lodz.pl (A.K.); mikolaj.malicki@stud.umed.lodz.pl (M.M.); 2 The Faculty of Medicine, Aichi Medical University, Nagakute 480-1195, Japan; wanibuchi.sora@gmail.com; 3 Department of Neurosurgery and Neuro-Oncology, Medical University of Lodz, Barlicki University Hospital, Kopcinskiego St. 22, 90-153 Lodz, Poland; michal.piotrowski@umed.lodz.pl (M.P.); ernest.bobeff@umed.lodz.pl (E.J.B.); dariusz.jaskolski@umed.lodz.pl (D.J.J.); 4 Department of Sleep Medicine and Metabolic Disorders, Medical University of Lodz, Mazowieka St. 6/8, 92-251 Lodz, Poland; 5 Department of Pediatrics, Oncology and Hematology, Medical University of Lodz, Sporna St. 36/50, 91-738 Lodz, Poland

**Keywords:** anterior inferior cerebellar artery, AICA, neurovascular compression syndromes, hemifacial spasm, geniculate neuralgia

## Abstract

The anterior inferior cerebellar artery (AICA) is situated within the posterior cranial fossa and typically arises from the basilar artery, usually at the pontomedullary junction. AICA is implicated in various clinical conditions, encompassing the development of aneurysms, thrombus formation, and the manifestation of lateral pontine syndrome. Furthermore, owing to its close proximity to cranial nerves within the middle cerebellopontine angle, AICA’s pulsatile compression at the root entry/exit zone of cranial nerves may give rise to specific neurovascular compression syndromes (NVCs), including hemifacial spasm (HFS) and geniculate neuralgia concurrent with HFS. In this narrative review, we undertake an examination of the influence of anatomical variations in AICA on the occurrence of NVCs. Significant methodological disparities between cadaveric and radiological studies (CTA, MRA, and DSA) were found, particularly in diagnosing AICA’s absence, which was more common in radiological studies (up to 36.1%) compared to cadaver studies (less than 5%). Other observed variations included atypical origins from the vertebral artery and basilar-vertebral junction, as well as the AICA-and-PICA common trunk. Single cases of arterial triplication or fenestration have also been documented. Specifically, in relation to HFS, AICA variants that compress the facial nerve at its root entry/exit zone include parabola-shaped loops, dominant segments proximal to the REZ, and anchor-shaped bifurcations impacting the nerve’s cisternal portion.

## 1. Introduction

### 1.1. Background on the AICA

The anterior inferior cerebellar artery (AICA) is one of the arteries located in the posterior cranial fossa. It originates from the basilar artery (BA), usually at the junction of the medulla oblongata and the pons. Its varied course in close proximity to cranial nerves’ root entry/exit zones (REZ) may affect the pathogenesis of a spectrum of disorders known as neurovascular compression syndromes (NVC), namely hemifacial spasm (HFS) and geniculate neuralgia. AICA aneurysms are rare and account for less than 1% of all intracranial aneurysms [1]. Arterial fenestration has been associated with an increased incidence of aneurysm formation [2]. Furthermore, AICA is associated with lateral pontine (Marie-Foix syndrome) syndrome, which consists of ipsilateral limb ataxia, loss of pain and temperature sensation of the face, facial weakness, hearing loss, vertigo, and nystagmus, as well as contralateral hemiplegia/hemiparesis and loss of pain and temperature sensation.

### 1.2. Neurovascular Compression Syndromes

NVCs are a group of conditions caused by vascular compression of the cranial nerves at REZ. This space, also known as Redlich–Obersteiner’s zone, is characterized by the transition from central to peripheral myelin. Therefore, it is presumably more vulnerable to mechanical factors, especially pulse waves [3]. The compression is speculated to be the cause of the onset of clinical symptoms; however, the exact pathological path remains unclear [4]. The proposed chain of pathogenetic events is vascular compression in the REZ/transition zone leading to demyelination; this causes nucleus hyperexcitability, which causes symptoms [4]. It is hypothesized that there might be another factor required, such as a previous minor injury, to trigger symptoms [5]. It is worth noting that compression is more often caused by arteries than veins, as the former pulsate and exert greater pressure [6].

Depending on the compressed REZ, various NVC manifestations are observed [7]. The most common are trigeminal neuralgia (TN) caused by trigeminal nerve compression, HFS, and glossopharyngeal neuralgia. In addition, single reports of superior laryngeal neuralgia as well as geniculate neuralgia associated with HFS symptoms can be found [3].

It has been proven that the frequency of individual manifestations of NVC is affected by embryological and anatomical variability [8,9], including variability of arteries [10]. In our work, we focused on anatomical variants of the AICA, leading to NVC.

### 1.3. Embryological Origins and Development of the AICA—A Concise Description

Lasjaunias proposed a comprehensive model for the embryological organization of blood supply to the posterior fossa. This model encompasses two distinct groups of arteries: longitudinal anterior (encompassing the basilar artery (BA) and the anterior spinal artery) and posterior (lateral medullary segment of the posterior inferior cerebellar artery (PICAs) and posterior spinal arteries) [11]. These arteries provide, among all, transverse arteries [11]. The embryological development of the AICA commences after the 44th day of embryogenesis. AICA, along with the superior cerebral artery (SCA) and the distal portion of the PICA, originate as a result of the fusion of transverse arteries. This fusion process leads to the establishment of connections through longitudinal posterior artery anastomoses [12].

### 1.4. Importance of Understanding Anatomical Variations of the AICA

**Figure 1:**
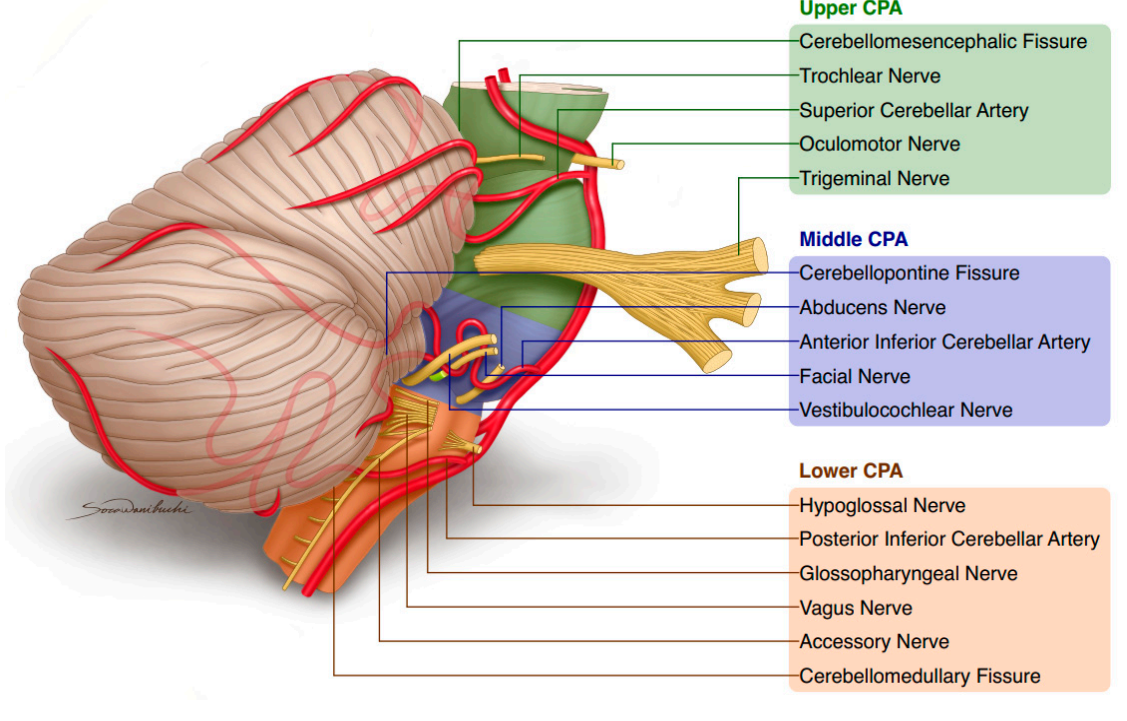
The anatomy of cerebellopontine angle (CPA) with graphical visualization of relation between anterior inferior cerebellar artery and root exit zone of facial nerve. Authors’ own figure prepared by Sora Wanibuchi. Its modified version was used in our previous publication in Biomedicines (mdpi.com).

The cerebellopontine angle (CPA) can be divided into the lower, middle, and upper parts (see Figure 1) [13]. The upper CPA comprises the cranial nerves: trigeminal, trochlear, oculomotor, and SCA. The middle CPA comprises the cranial nerves: abducens, facial, vestibulocochlear, and anterior inferior cerebellar artery (AICA). The inferior part comprises the cranial nerves such as the hypoglossal, glossopharyngeal, vagus, accessory, and posterior inferior cerebellar artery (PICA) [14].

AICA, located in the middle CPA, is associated with HFS due to its conflict with the facial nerve and single with geniculate neuralgia from the nervus intermedius. Moreover, there are papers suggesting its significance in disabling positional vertigo. Nowadays, this condition (similar to arterial hypertension in the course of NVC at the glossopharyngeal/vagus nerve and torticollis) should not be classified as an NVC-related state, as there is insufficient clinical evidence for the effectiveness of microvascular decompression (MVD) in those cases. All these clinical manifestations of AICA-triggered NVC syndromes raise a question about the importance of their anatomical variability [10,15,16].

## 2. Anatomy of the AICA

AICA is one of the arteries located in the posterior cranial fossa that usually originates as a single trunk on the lateral wall of the lower or middle third of the BA at the pontine level. Depending on the source, the AICA diameter is typically between 0.9 and 1.4 mm [17]. AICA is generally the most caudal branch of BA [17,18]. Then, it runs posterolaterally and inferiorly [19], ventral to the pons. It enters the CPA and courses along the anterior cerebellar surface. The course of AICA is tortuous. Therefore, its relation to cranial nerves may vary: AICA may loop around the facial and vestibulocochlear nerve trunks superiorly or inferiorly or pass in between them. Examples of AICA looping between the facial nerve and nervus intermedius were reported [20,21,22]. Cadaver studies describe loops of AICA that reach into the internal acoustic meatus [20,21].

The AICA bifurcates into the upper and lower trunks at the pontomedullary junction adjacent to the exit of the facial and vestibulocochlear nerves from the brainstem [22]. AICA is divided into four segments: anterior pontine (close to the abducens nerve), lateral pontine (close to the facial and vestibulocochlear nerves), flocculopeduncular, and cortical. The nerve-related AICA trunks are further divided into three segments based on their relationship to the internal auditory meatus: the premeatal, meatal, and postmeatal segments. The meatal segment often projects into the auditory canal [23] and contains the origin of the labyrinthine artery.

The supply area of AICA includes the middle cerebellar peduncle, the anterolateral portion of the pons, the superolateral part of the medulla oblongata, the anteroinferior part of the cerebellum, the flocculus and the choroid plexus at the lateral aperture of the fourth ventricle. AICA usually supplies the following nuclei: trigeminal, facial, vestibular, and cochlear [19,24]. The labyrinthine artery supplies the peripheral audiovestibular structures of the inner ear, along with the facial and vestibulocochlear nerves [25,26].

## 3. Neurovascular Compression Syndromes Related to the AICA

### 3.1. Hemifacial Spasm

Clinically, HFS is a disorder defined as intermittent twitching of facial muscles caused by pulsating compression exerted on the REZ of the facial nerve by an artery, usually the AICA. Its average incidence is about 10 per 100,000 [27,28]. Typically, HSF presents as involuntary, usually unilateral clonic and/or tonic spasms/contractions of the facial expression muscles [29,30]. Uncontrolled spasms usually start from the orbicularis oculi muscle with further, gradual involvement of other muscles, while atypical HFS starts from the spasm of the muscular orbicularis oris, gradually spreading upward to involve the orbicularis occuli muscle [31,32]. Various manifestations result from different patterns of facial nerve compression—the anterior/caudal aspect for a typical HFS and the posterior/rostral aspect for an atypical HFS [32].

Pathophysiological investigations carried out by Moller et al. in patients afflicted with HFS appear to substantiate the involvement of the facial motonucleus as a secondary consequence arising from compression of the REZ, a phenomenon akin to kindling [33]. Furthermore, their research delineates an additional electrophysiological phenomenon, denominated synkinesis, wherein the stimulation of one branch of the facial nerve elicits delayed discharges through another branch [33,34].

HFS may be caused by the following arteries: AICA (37%), posterior inferior cerebellar artery (PICA; 30%), and VA/BA (23%); in other cases, facial nerves may be compressed by veins [35]. Furthermore, HFS differential diagnosis encompasses infections, CPA tumors, brainstem lesions, some cases of Bell’s palsy, blepharospasm, facial myokymia, etc. [36,37,38].

### 3.2. Geniculate Neuralgia

Identifying the primary source of otalgia can present a diagnostic challenge [39,40] due to the complex sensory innervation of the auditory canal, encompassing not only the nervus intermedius but also the trigeminal, glossopharyngeal, and vagus nerves. One of the entities is geniculate neuralgia, also known as Hunt’s neuralgia. It is characterized by intense paroxysms of stabbing pain localized inside the ear [39]. The pain is triggered by various sensory stimuli, including exposure to coldness and noise. Although it is mainly related to a herpes zoster infection of the geniculate ganglion, selected cases result from compression of the nervus intermedius [16]. In the second scenario, geniculate neuralgia coexists with HFS symptoms. Unsurprisingly, only in these cases may MVD be considered.

### 3.3. Disabling Positional Vertigo

Given the present scope of medical understanding, disabling positional vertigo should not currently be categorized as a condition related to neurovascular compression, akin to arterial hypertension in NVC at the glossopharyngeal/vagus nerve or torticollis. The justification for this is the inadequate clinical evidence supporting microvascular decompression as a therapeutic intervention for this specific manifestation [4]. Therefore, this subchapter has mainly historical significance.

Disabling positional vertigo is a specific syndrome previously described as an effect of compression of the vestibular nerve or vestibulocochlear complex by a blood vessel. This pressure causes patients to suffer from constant positional vertigo and nausea to the extent that their life quality is severely lowered [41]. Previous studies suggested that it may result from the compression of the vestibulocochlear nerve, e.g., by the AICA loop extending into the internal auditory canal and compressing the vestibulocochlear nerve [15].

## 4. Anatomical Variations of the AICA

Cerebellar arteries are characterized by high variability; it is estimated that only 11.7% remain “anatomically unchanged” [42]. The prevalence of the anterior inferior cerebellar artery anatomical variants is collected in Table 1. Differences between studies may be explained by different methodologies: cadaveric studies [18,21,23,26,43] vs. radiological studies (computed tomography angiography (CTA) [42,44], magnetic resonance angiography (MRA) [44], and digital subtraction angiography (DSA) [19]). The absence of AICA was frequently diagnosed in CTA, MRA, and DSA, but not in cadaver studies [45]. Some authors divide the BA site of AICA origin into three parts (upper third, middle third, and lower third of BA), while others use two-part nomenclature (upper and lower part of BA). Finally, authors differ in the method of percentage calculation: the study group size was sometimes presented as the number of whole brain specimens (or examined patients), while in other works it was presented as the number of examined hemispheres.

The most common variations visualized using radiological imaging are: absence (3.1–36.1%), duplication (7.9–10.4%), and atypical origin site (9.4%). The most common variations confirmed in anatomical studies are: duplication (22–39%), atypical origin site (2.8–47%), and absence (up to 4%; see further subchapters for detailed information).

### 4.1. Agenesis

Regarding radiological studies (CT, DSA, MRI), the agenesis of AICA is observed in up to 36.1% of cases: 5.6–11.1% for right, 11.1–16.1% for left [42,44], and 6.7–14.4% for both sides [42,43,44]. In contrast, in cadaveric studies, it was observed in less than 5%; the bilateral AICA agenesis was not described in these cases [18,21,23,26,43,45]. These discrepancies may be explained by: imperfection of angiography in the case of imaging very small vessels; and different study group sizes (greater in radiological studies).

The simultaneous absence of both AICA and PICA has not been documented [44], suggesting a compensatory relationship between these vessels. When one artery is rudimentary, the other becomes dominant, a phenomenon termed the AICA-PICA reciprocal arrangement [19]. This hypothesis posits an inverse proportionality between AICA’s size and supplied territory and those of PICA. Notably, elongated AICA, extending to the cerebellum’s posterior inferior surface, often coincides with PICA’s atresia or absence [43].

### 4.2. Atypical Origin Site

The literature describes the following origin sites of AICA: BA, vertebral artery (VA), and the BA-VA junction [19]. Furthermore, cases of an AICA-and-PICA common trunk extend from VA (type V) or BA (type B), and the branches are named accordingly to their supply area [19].

### 4.3. Other Variants

Single studies describe arterial triplication [23,46] or fenestration [42]. Fenestration is the division of the arterial lumen, resulting in two separate channels (each with its own endothelial and muscular layer) that connect on both ends. Clinically, it is associated with an increased incidence of aneurysm formation [2].

## 5. Clinical Significance of Anatomical Variations of the AICA

### 5.1. Relationship with Neurovascular Compression Syndromes

**Figure 2:**
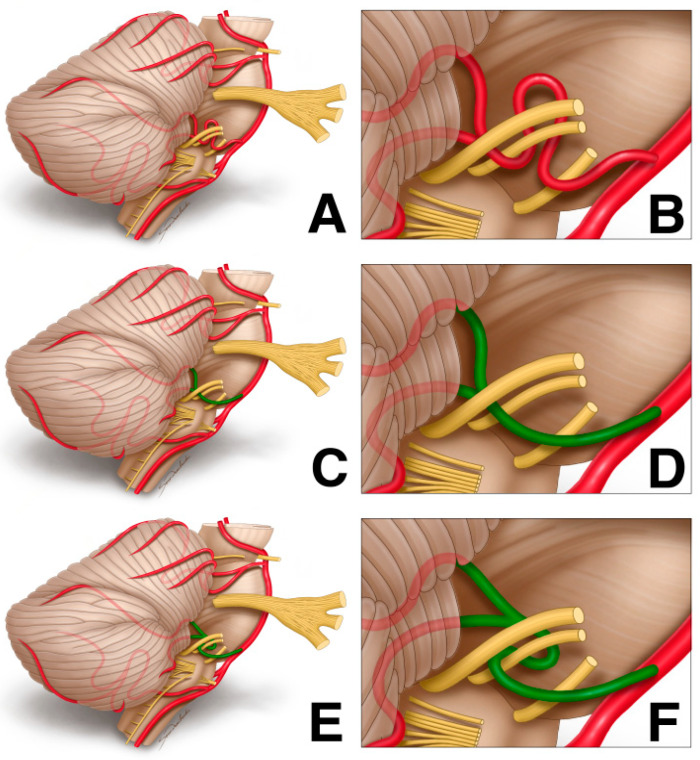
AICA courses described in HFS patients based on patterns, in which AICA is passing between the facial and vestibulocochlear nerves: (**A**,**B**) a parabola-shaped loop that is vertex-oriented to the facial nerve’s REZ; (**C**,**D**) a large dominant AICA segment proximal to the REZ; (**E**,**F**) an anchor-shaped AICA bifurcation which affects the cisternal portion of the facial nerve (see Li et al. for further details).

To date, there is no clear evidence linking selected AICA variants to a higher risk of NVC incidence. Therefore, we decided to look at AICA courses described in HFS patients based on a pattern that AICA takes while passing in between the facial and vestibulocochlear nerves: (1) a parabola-shaped loop that is vertex-oriented to the facial nerve’s REZ; (2) a large dominant AICA segment proximal to the REZ; and (3) an anchor-shaped AICA bifurcation that affects the cisternal portion of the facial nerve (see Figure 2) [47].

### 5.2. Other Relationships with Cranial Nerves (nonNVC in Its Nature)

Intraoperative findings show that AICA can impinge on the facial nerve near the internal auditory canal [42]. These findings should not be considered a true HFS caused by the compression on the facial nerve REZ [31,47,48,49,50,51].

Smaller case series suggest that AICA is crossing over the gap between the facial nerve and the cochleovestibular complex at the cisternal portion, resulting in compression of the facial nerve distally to the REZ. Another situation is AICA penetrating through several bundles of the facial nerve, also in the cisternal portion [31]. Distal compression can also occur in the internal auditory meatus. There, the loop of the meatal segment of AICA can induce a cross-type compression by passing through the facial vestibulocochlear nerves [50].

### 5.3. Aneurysm Development and Thrombus Formation

AICA aneurysms are a rare condition that account for less than 1% of all intracranial aneurysms [2]. They may also lead to brainstem compression, trigeminal, abducens, facial, and vestibulocochlear nerve neuropathies, mass effect, hearing loss, tinnitus, facial spasm, diplopia, and paresthesia within the trigeminal nerve innervation area [52,53,54].

The location and prevalence of AICA aneurysms vary between publications (see Table 2). Typically, aneurysms occur in the premeatal part of the AICA or proximally (i.e., either in the premeatal segment or at the basilar-AICA junction) and rarely in the meatal part. The least frequent location is the distal or postmeatal AICA’s portion [1,53,55,56,57].

Each location produces characteristic clinical symptoms. Thus, proximal aneurysms are more commonly associated with subarachnoid hemorrhage, brainstem compression, progressive headaches, dizziness, and the abrupt onset of trochlear and abducens nerve palsies [2,58,59], whereas distally located aneurysms may present with trigeminal neuralgia, tinnitus, vertigo, deafness, headache, and vomiting [54,60,61,62,63].

Ischemic stroke in the distribution of AICA remains a rare condition, accounting for up to 3% of strokes. Predisposing risk factors include hypertension, diabetes mellitus, hyperlipidemia, atrial fibrillation, prior transient ischemic attack, and previous strokes [64,65,66]. In the vast majority of cases, the stroke is unilateral; bilateral involvement results more from embolism than thrombosis [67].

The AICA’s supply territory is a home to around 12% of cerebellar infarctions, many of which go misdiagnosed [66,67]. The symptoms depend on the anatomical course of the artery and the exact area supplied by AICA. Proximal branches of AICA supply blood to the posterolateral pons: cranial nerve nuclei (trigeminal, facial, vestibulocochlear) and fascicles (facial, vestibulocochlear), sympathetic tract, spinothalamic tract, and middle cerebellar peduncle. The distal part of AICA provides blood to the anteroinferior cerebellum, flocculus, and inner ear, including the vestibular labyrinth and cochlea [64,66]. Patients present non-specific symptoms like dizziness, nausea and vomiting, gait instability, and headaches. The characteristic sign of acute vestibular syndrome is hearing loss following a deficit of blood supply to the labyrinthine artery, the vestibular nuclei and fibers, or the flocculus [62,66,68].

### 5.4. Lateral Pontine Syndrome

Lateral pontine syndrome results from a brainstem stroke encompassing the lateral pons. Clinically, the pons can be divided into three vascular territories: the anteromedial territory, the anterolateral territory, and the lateral territory [69]. The final two are partially or entirely supplied by AICA [70]. The following symptoms are characteristic: ipsilateral limb ataxia, loss of pain and temperature sensation from the face, facial weakness, hearing loss, vertigo, and nystagmus, as well as contralateral hemiplegia/hemiparesis and loss of sensation to pain and temperature [69]. It is worth emphasizing that AICA territory infarcts are not only very rare, but also one of the most challenging to demonstrate in neuroimaging [66].

## 6. AICA Course in Radiological and Intraoperative Examination

Neuroimaging techniques, especially MRI, are employed in NVC patients to facilitate differential diagnosis, enabling the exclusion of alternative pathologies such as CPA tumors, pontine gliomas, cerebral aneurysms, or arteriovenous malformations [71]. However, the MVD’s success rate depends on intraoperative visualization of the cranial nerve in its REZ, not on previous neuroimaging [72]. In essence, patients presenting with NVC’s symptoms in whom appropriate pharmacological treatment fails are good candidates for MVD, even if NVC was not detected in the imaging studies [4]. The subsequent subsections address various topics of relatively restricted relevance to direct clinical practice.

### 6.1. Importance of Understanding Anatomical Variations in Diagnosis and Surgical Treatment

High-resolution MR images provide reliable and detailed information on the corresponding intraoperative anatomy [73]. Specific MRI methods useful for imaging NVCs include three-dimensional magnetic resonance (3DMR), while the vertebrobasilar circulation variants may be visualized using CTA, MRA [44], and DSA [19].

#### 6.1.1. Magnetic Resonance Angiography

MRA is a common term encompassing different types of MRI technology, employed specifically for the visualization and assessment of blood vessels. This technique utilizes the principles of MRI but adapts them to focus on the unique characteristics of blood flow and vascular structures, offering a distinct and specialized approach to vascular imaging, with or without contrast enhancement. The latter one can be further divided into: flow independent (balanced steady-state free precession), non-subtractive inflow-dependent (including: time of flight angiography, TOF, inflow-dependent inversion recovery, quiescent interval slice-selective), phase contrast angiography, and subtractive 3D MRA (including: cardiac-gated 3D fast spin-echo, flow-sensitive dephasing, and arterial spin labeling). In the context of NVC detection, TOF MRA seems to be the most appropriate approach [74]. This non-contrast approach bases itself on the natural contrast generated by blood flow. When unsaturated blood flows into an imaging slice, its spins emit a stronger signal compared to the surrounding, saturated tissue spins. Finally, blood vessels are brighter on the images, allowing for clear and detailed visualization of vascular structures. Clinically, it provided supportive evidence of surgical indications in patients with TN and HFS, although attention should be paid to the fact that MRA did not necessarily detect all of the offending vessels [75].

#### 6.1.2. Three-Dimensional Magnetic Resonance

The preferred method for visualizing NVC in neuroimaging is the application of three-dimensional constructive interference in steady state (CISS), also referred to as fast imaging employing steady state acquisition (FIESTA) or true fast imaging with steady-state free precession (TRUFI) [76].

These particular MRI sequences are an example of fully refocused fast gradient echo sequences, featuring both pre- and post-excitation signals, yielding a substantial signal-to-noise ratio and exceptional spatial resolution. The acquisition process involves the recycling of the decaying signal originating from the transverse magnetic plane, facilitated by the application of a 180-degree phase shift during each repetition time interval. Concurrently, there is a transition of the residual transverse magnetization field, transitioning from the Z-axis to the longitudinal X-axis. After numerous repetition cycles, an equilibrium state is achieved in both the transverse and longitudinal planes. In practice, this imaging technique furnishes excellent contrast differentiation among cranial nerves, small vessels, and cerebrospinal fluid [77].

An alternative approach to medical imaging involves the application of high-resolution 3DMR cisternography and virtual endoscopy (VE). These imaging modalities offer utility in specific clinical contexts, and thus, we present a succinct overview of their applicability. Firstly, high-resolution 3DMR cisternography techniques are employed in the preoperative assessment of neurovascular anatomy. However, they often exhibit limitations in achieving adequate contrast differentiation between blood vessels and cranial nerves precisely at the site of the NVC. To address this limitation, a method known as Subtraction of 3D T2-weighted Imaging-driven Equilibrium Radiofrequency Reset Pulse from Contrast-enhanced 3D T1-weighted Imaging has been empirically demonstrated to provide both superior spatial resolution and outstanding contrast enhancement for depicting neurovascular compression [78]. Secondly, another valuable technique in three-dimensional MRI visualization is VE. Presurgical implementation of VE in patients with NVCs’ patients offers exceptional visualization of the dimensional relationships among neurovascular structures. Furthermore, VE enables the simulation of MVD procedures, thereby enhancing surgical planning and decision-making processes [79].

#### 6.1.3. CTA and DSA in NVC Patients

Vertebrobasilar circulation variants can be visualized not only through the previously mentioned MRA techniques but also in CTA and DSA [19]. CTA, particularly of the circle of Willis, is recognized for its wide availability, speed, low-invasiveness, and cost-efficiency in visualizing intracranial arteries. Despite its advantages, the primary limitation of CTA is the necessity for contrast agent administration, which might not be suitable for all patients.

On the other hand, DSA is an invasive technique that is considered the gold standard for diagnosing intracranial artery diseases. This modality requires the administration of an iodine-based contrast medium, akin to one employed in CTA. The precision and detail offered by DSA make it invaluable, especially in complex cases. These imaging techniques are typically recommended for conditions such as suspicion of intracerebellar aneurysms, subarachnoid hemorrhage, and arteriovenous malformations.

However, it is important to note that in patients with NVC, these imaging methods are not generally indicated, as in the vast majority of NVC cases, their findings would not alter the treatment approach. The utilization of these imaging modalities exposes the patient to radiation and contrast agents without offering significant advantages. Consequently, the decision to utilize these imaging techniques should be carefully considered, weighing potential benefits against risks, especially given the invasive nature of DSA and the need for contrast in CTA.

### 6.2. Intraoperative Findings

In this chapter, we present a surgical perspective on AICA variations in NVC patients, based on review articles and an extensive surgical series of MVD.

Mercier and Sindou carried out an ambitious project aiming to assess the epidemiology of conflicting vessels in HFS [48]. They combined a thorough literature review with an analysis of their own two series of 2489 and 340 patients. They discovered that the most common artery responsible for the conflict was PICA (47.2%), followed by AICA (45.9%), the vertebro-basilar complex (17.5%), and other smaller arteries (11.7%), and veins (4.9%). Unsurprisingly, multiple vascular compressions of the facial nerve were noted in 20–37% of patients [48].

The authors proposed four distinct zones of the facial nerve for surgical exploration, drawing on the frameworks provided by Campos-Benitez, Lijima, and the Shanghai team. These zones include the nerve root exit point/zone, the attached segment (also known as the root emerging zone), the nerve root detachment point, and the cisternal portion. They suggested that exploring all the zones could account for the variability in the frequency of the identified compressing vessels.

Furthermore, Mercier and Sindou emphasized the importance of understanding anatomical variants to accurately identify the source of conflict and, consequently, to perform effective and safe MVD. They pointed out that particular challenges might arise in patients with arteriosclerotic megadolicho-vertebrobasilar complex anomalies impacting the brainstem, underscoring the need for meticulous surgical planning and execution in these complex cases.

Teton et al. used an innovative application of 3D imaging reconstructions to assess symptom-free patients who underwent MVD in HFS. Their retrospective study encompassed 35 patients, revealing that the most common site of culprit compression typically occurs proximally along the brainstem, specifically at the attached segment [80]. Thus, Teton et al. advocate the utilization of three-dimensional imaging reconstructions in the preoperative phase not only to precisely identify suitable surgical candidates but also to guide the planning of operative strategies in order to improve the surgical outcome [80]. They believe that by integrating 3D imaging into the pre-surgical workflow, surgeons gain a comprehensive understanding of the anatomical landscape, which makes the operation easier. Hence, this methodology might be generally helpful in improving the efficacy of MVD in terms of clinical outcome [80].

In a pioneering effort, Pham et al. conducted a prospective cross-sectional study to develop a classification system based on intraoperative endoscopic views of NVC in HFS patients [81]. Analyzing data from 29 HFS patients who underwent endoscopy-assisted MVD and experienced no postoperative symptoms, they made several key observations. The REZ emerged as the most frequent site of NVC, with the AICA being the predominant compressing vessel [81]. Interestingly, the most common form of NVC was identified as the vascular loop.

They encourage the use of a classification encompassing six types of nerve compression previously described by Park et al. [81,82]:

*Loop*: compression solely by the vascular loop.

*Arachnoid*: compression occurs when thick arachnoid trabeculae tether the vessel tightly to the nerve.

*Perforator*: characterized by perforating arteries from the compressing vessel, causing them to be tethered to the brainstem.

*Branch*: the nerve is caught between the compressing vessel and its branch.

*Sandwich*: the nerve is sandwiched between two different vessels independently.

*Tandem*: one vessel compresses another, which then compresses the nerve.

## 7. Conclusions

The prevalence of anatomical AICA’s variants varies between publications, which may be an effect of different methodologies (cadaveric vs. radiological studies), the small size of the study group, and the different populations studied. Studies encompassing a substantial sample size, exceeding 100 cases, are rare. Furthermore, the utilization of different nomenclature affects reliable comparative analysis in these studies. Consequently, additional investigations elucidating AICA’s anatomical variations are legitimate.

In summary, our examination of AICA’s implications for HFS and geniculate neuralgia has yielded the following considerations:

- The AICA emerges as the main vascular structure associated with the pathogenesis of HFS. Additionally, in some cases, it can contribute to the manifestation of geniculate neuralgia alongside HFS symptoms.
- Neuroimaging plays a crucial role in facilitating accurate differential diagnosis by excluding other central nervous system pathologies such as CPA tumors, pontine gliomas, aneurysms, or arteriovenous malformations.
- The MVD’s success rate strictly depends on meticulous intraoperative visualization of a compressing vessel on the nerve within its REZ during surgery, not solely on radiological examination.
- HFS is characterized by intermittent twitching of facial muscles, initiating from the orbicularis oculi muscle, with downward progression involving other facial muscles. A prominent feature is the involuntary, typically unilateral, clonic and/or tonic spasms and contractions of facial expression muscles.

## Figures and Tables

**Table 1:** Prevalence of the anterior inferior cerebellar artery anatomical variations. Studies were group by approach (cadaver and radiological studies) and publication year.

		Cadaver Studies					Radiological Studies		
		Scialfa et al. (1976) N = 30 ^a^ [43]	Naidich et al. (1976) N = 32 ^a^ [21]	Martin et al. (1980) N = 50 ^b^ [23]	Pai et al. (2007) N = 25 ^a^ [40]	Delion et al. (2016) N = 25 ^a^ [26]	Akgun et al. (2013) N = 135 ^c^ [44]	Y. and R. Pekcevik (2014) N = 341 ^c^ [42]	Hou et al. (2019) N = 1000 ^d^ [19]
Agenesis	total	3.3% ^e^	N.D.	N.D.	0%	4%	28.9%	36.1%	3.1%
	right	N.D.	N.D.	N.D.	N.D.	N.D.	11.1%	5.6%	N.D.
	left	N.D.	N.D.	N.D.	N.D	N.D.	11.1%	16.1%	N.D
	bilateral	0%	N.D.	N.D.	N.D	N.D.	6.7%	14.4%	N.D
Duplication	total	26.7%	29%	26%	22%	N.D.	N.D.	7.9%	10.4%
	right	N.D.	N.D.	N.D.	N.D	N.D.	N.D.	5.3%	N.D.
	left	N.D.	N.D.	N.D.	N.D	N.D.	N.D.	2.3%	N.D.
	bilateral	10%	N.D.	N.D.	N.D.	N.D.	N.D.	N.D.	N.D.
Triplication		N.D.	1%	2%	N.D.	N.D.	N.D.	N.D.	0%
Fenestration		N.D.	N.D.	N.D.	N.D	N.D.	N.D.	0.3%	N.D
Origin site	the upper third of BA	1.4%	47%	N.D.	32.1%	24% (upper half) 75% (lower half)	N.D.	N.D.	0.8% (upper half) 90.6% (lower half)
	the middle third of BA	0%	53%	N.D.	60.7%		N.D.	N.D.	
	the lower third of BA	97.1%	0%	N.D.	7.1%		N.D.	N.D.	
	BA-VA junction	N.D.	0%	N.D	N.D.	9% ^f^	N.D.	N.D.	4.3%
	VA	1.4%	0%	N.D.	N.D.	N.D.	N.D.	N.D.	4.3%

Legend: a—whole brain specimens, cadaver studies, b—hemisphere specimens, cadaver studies, c—whole brain, ante mortem imaging (CT, MR), d—hemispheres, ante mortem imaging (DSA), e—unilateral, f—described as ”close to the junction”; BA—basilar artery, N.D.—no data, VA—vertebral artery.

**Table 2:** The frequency of anterior inferior cerebellar artery aneurysms depends on the location.

	Distal/Postmeatal	Proximal/Premeatal	Meatal	BA-AICA Junction
Drake et al. (1996) [55]	4 (9.5%)	4 (9.5%)	N.D.	33 (80%)
Gonzalez et al. (2004) [1]	4 (11.8%)	30 (88.2%)	N.D.	N.D.
Sanai et.al. (2008) [57]	6 (75%)	2 (25%)	N.D.	N.D.
Li et al. (2012) [56]	2 (33.3%)	1 (16.7%)	3 (50%)	N.D.
Lvet al. (2016) [53]	10 (21.3%)	16 (34%)	21 (44.7%)	N.D.

**Legend:** AICA—anterior inferior cerebellar artery, BA—basilar artery, N.D.—no data.

## Abbreviations

3DMR	three-dimensional magnetic resonance
AICA	anterior inferior cerebellar artery
BA	basilar artery
CPA	cerebellopontine angle
CTA	computer tomography angiography
DSA	digital subtraction angiography
FIESTA	fast imaging employing steady state acquisition
HFS	hemifacial spasm
MRA	magnetic resonance angiography
MVD	microvascular decompression
NVC	neurovascular compression syndromes
PICA	posterior inferior cerebellar artery
REZ	root entry/exit zone
TN	trigeminal neuralgia
TOF	time-of-flight magnetic resonance angiography
TRUFI	true fast imaging with steady-state free precession
VE	virtual endoscopy
